# Integrating Network Analysis and Machine Learning Identifies Key Autism Spectrum Disorder Genes Linked to Immune Dysregulation and Therapeutic Targets

**DOI:** 10.3390/genes16091109

**Published:** 2025-09-19

**Authors:** Haitang Wang, Xiaofeng Zhu, Hong Zhang, Weiwei Chen

**Affiliations:** Pinghu Normal College, Jiaxing University, Jiaxing 314041, China

**Keywords:** PPI network, random forest, immune infiltration, biomarker

## Abstract

Background: Understanding the genetic mechanisms and identifying potential therapeutic targets are essential for clarifying Autism Spectrum Disorder (ASD) etiology and improving treatments. This study aims to bridge the gap between basic transcriptomic discoveries and clinical applications in ASD research. Methods: Differentially expressed genes (DEGs) of GSE18123 datase were identified. A protein–protein interaction (PPI) network was constructed. Functional enrichment analysis was performed to link genetic loci to relevant biological pathways. Connectivity Map (CMap) analysis was used to predict potential drugs. Furthermore, immune infiltration correlation analysis explored associations between key genes and immune cell subpopulations. Diagnostic performance of top genes was evaluated by receiver operating characteristic (ROC) analysis. Results: The functional enrichment analysis successfully revealed relevant biological processes associated with ASD, while the CMap analysis predicted potential drugs that were consistent with some clinical trial results. Random forest analysis selected ten key feature genes (*SHANK3*, *NLRP3*, *SERAC1*, *TUBB2A*, *MGAT4C*, *TFAP2A*, *EVC*, *GABRE*, *TRAK1*, and *GPR161*) with the highest importance scores for autism prediction. Immune infiltration analysis showed significant correlations in genes and multiple immune cell types, demonstrating complex pleiotropic associations within the immune microenvironment. ROC curve analysis indicated that most top genes had strong discriminatory power in differentiating ASD from controls, particularly MGAT4C (AUC = 0.730), highlighting its potential as a robust biomarker. Conclusions: This study effectively bridges the basic transcriptomic discoveries and clinical applications in ASD research. The findings contribute to a better understanding of the etiology of ASD and provide potential therapeutic leads. Future research could focus on validating these potential drugs in clinical studies, as well as further exploring the biological functions of the identified genes to develop more targeted and effective treatments for ASD.

## 1. Introduction

Autism Spectrum Disorder (ASD) is a neurodevelopmental disorder characterized by high clinical and genetic heterogeneity. Its core features are persistent deficits in social communication and social interaction, alongside restricted, repetitive patterns of behavior, interests, or activities. According to current international authoritative diagnostic criteria (such as DSM-5 and ICD-11), ASD is defined as a continuous “spectrum” disorder [1,2]. This definition supersedes previous subcategories based on symptom presentation and functional levels, emphasizing the continuous dimensional variation in symptoms ranging from mild to severe. The diagnosis of ASD is primarily based on behavioral observations and developmental history assessment, requiring comprehensive judgment by specialized clinicians using standardized tools. It is typically identifiable in early childhood (ages 2–3 years), although significant individual variations exist in the age of diagnosis and symptomatic manifestations [3]. The present study aims to further explore the potential pathological mechanisms of ASD at the molecular level, thereby providing initial insights that could contribute to the long-term goal of precision medicine.

Recent research has yielded significant advances in deeply unraveling the etiology and pathophysiology of ASD. Diverse research approaches are progressively advancing our understanding of this complex condition across multiple dimensions and hierarchical levels. In the molecular genetics domain, large-scale genome-wide association studies (GWAS), whole-exome sequencing (WES), and copy number variation (CNV) analyses have identified hundreds of genetic loci significantly associated with ASD risk (*SHANK3*, *NLGN3/4*, *CHD8*), revealing abnormalities in key biological pathways involved in synaptic function, chromatin remodeling, and transcriptional regulation [4,5]. Neurobiological mechanism research increasingly relies on advanced multimodal neuroimaging techniques (fMRI, sMRI, dMRI), delineating abnormal early developmental trajectories of brain structure and functional connectivity networks in children with ASD [6].

Cellular and animal model studies utilize neurons/glia differentiated from induced pluripotent stem cells and genetically engineered animal models to mimic specific genetic defects through in vitro and in vivo experiments, deeply exploring ASD-related cellular behavioral alterations and identifying potential therapeutic targets [7,8]. Epidemiological and comorbidity studies focus on associations between environmental risk factors and ASD prevalence, while systematically analyzing the impact of common co-occurring disorders on individual functional outcomes [9]. Clinical translation and intervention research is dedicated to developing precision assessment tools and combined behavioral–pharmacological strategies to optimize individualized treatment plans [10]. Although the aforementioned research has substantially expanded the boundaries of our knowledge regarding ASD, the mechanisms for integrating and translating findings across these different levels of investigation remain incompletely elucidated. A critical challenge persists in establishing the causal links from genetic variants and aberrant neural circuitry to observable behaviors within the complex interactions of the biological system. Although research in cellular and animal models, epidemiology, comorbidity, and clinical translation has greatly expanded our knowledge of ASD, the mechanisms for integrating and translating findings across different levels of investigation remain incompletely understood. A major challenge lies in establishing the causal links from genetic variants and aberrant neural circuitry to observable behaviors within the complex interactions of the biological system.

By bridging the gap between basic transcriptomic discoveries and clinical applications, this work aims to contribute both mechanistic insights into ASD etiology and tangible leads for therapeutic development. The integration of multiple analytical dimensions from single gene differential expression to system level network pharmacology represents a significant methodological advance over previous fragmented approaches to ASD transcriptomics.

## 2. Materials and Methods

### 2.1. Data Acquisition and Preprocessing

The human autism and normal control blood sample microarray dataset GSE18123 was retrieved and downloaded from the National Center for Biotechnology Information (NCBI)/GEO [11]. From the GEO series GSE18123 (285 peripheral blood samples profiled on two platforms: GPL570 and GPL6244) comprising multiple diagnostic categories (ASD, Pervasive Developmental Disorder–Not Otherwise Specified [PDD-NOS], Asperger’s Disorder, and Controls), we restricted analyses to a homogeneous, single-platform subset to minimize diagnostic and technical heterogeneity. Specifically, we selected the GPL570 subset (99 samples) and included only those labeled as ASD and Control (31 ASD, 33 Control), excluding PDD-NOS and Asperger’s Disorder (*n* = 36) and all samples from GPL6244. This predefined filtering ensured a clear binary classification task (ASD vs. Control) and reduced potential confounding from cross-platform effects and mixed neurodevelopmental diagnoses.

The raw data were generated using the Affymetrix Human Genome U133 Plus 2. 0 Array platform (Affymetrix, Santa Clara, CA, USA) (GPL570), including the autism group (*n* = 31) and the control group (*n* = 33). R software (version: 4.2.2) and relevant Bioconductor packages (such as limma, version: 3.58.1, affy, version: 1.80.0) were used for background correction, normalization, and batch effect removal of the original expression matrix.

### 2.2. Differentially Expressed Gene (DEG) Analysis

Differential analysis between the autism and control groups was performed using the “limma” R package (version: 3.58.1) and a linear modeling approach. The screening criteria were |log2FC| > (1.5) and adjusted *p*-value (FDR) < 0.05. The results were visualized as volcano plots and heatmaps.

### 2.3. DEG Functional Enrichment Analysis 

Gene Ontology (GO)/Kyoto Encyclopedia of Genes and Genomes (KEGG) [12,13]. Pathway enrichment analysis was conducted on the DEGs using the clusterProfiler R package (version: 4.10.1) and others, covering biological process (BP), molecular function (MF), and cellular component (CC) categories. Hypergeometric distribution was used for statistical testing (*p* adjust. method = “BH”, significance threshold *p* < 0.05). Enrichment results were displayed in a chord diagram and similar visualization forms. The DEG interaction network was obtained via the STRING database (https://string-db.org, confidence score threshold ≥ 0.4) and imported into Cytoscape software (version: 3.10.3) for visualization [14,15,16,17]. The confidence threshold of 0.4 in the PPI analysis using STRING refers to the combined score, which indicates that this value is effective in filtering out low confidence interactions while retaining a sufficient number of relevant connections for our analysis.

### 2.4. CMap Drug Prediction

Upregulated and downregulated DEGs were submitted to the Connectivity Map (CMap) online platform (https://clue.io) for drug reversal prediction [18,19]. Candidate drugs with the top (6) enrichment scores were identified as potential regulatory molecules.

### 2.5. GeneCard Disease-Related Gene Retrieval and Downstream Analysis

Genes related to autism (Autism Spectrum Disorder) were retrieved from the GeneCard database (https://www.genecards.org/), using a high relevance score threshold (score > 10) [20,21]. These were intersected with the DEGs to yield candidate key genes. The intersection genes were subjected to GO enrichment and PPI network analysis as described above.

### 2.6. Screening for Feature Genes Using Random Forest

Data were randomly divided into training (70%) and validation (30%) sets. Random forests were trained using the R randomForest package (version: 4.7-1.2) with ntree = 500 and nPerm = 5. OOB estimates were utilized to calculate MeanDecreaseGini importances [22]. Genes were ranked by MeanDecreaseGini; the ten highest-ranked genes were designated as the “Top 10” feature set. Predictive performance was assessed using OOB error on the training set and held-out validation metrics (confusion matrix and ROC/AUC computed from predictions on the validation set).

### 2.7. Immune Landscape Analysis

Immune deconvolution analysis was performed using the R package “GSVA” (version: 1.46.x) to resolve the transcriptomic expression matrix into constituent proportions of diverse immune cell subtypes. Associations between the top-ranked ten pivotal genes and patterns of immune cell infiltration were quantitatively evaluated via Spearman or Pearson correlation analysis. Statistically significant correlations (*p* < 0.05) were visually represented through correlation heatmaps constructed with the “corrplot” R package (version: 0.95).

### 2.8. ROC Curve Analysis

The “pROC” R package (version: 1.19.0.1) was used to calculate the area under the curve (AUC) of the top 10 feature genes and to assess their diagnostic value in ASD screening. An AUC greater than 0.7 was considered indicative of good discriminative ability. Visualizations included both single-gene and combined ROC curves.

## 3. Results

### 3.1. Differentially Expressed Gene (DEG) Screening Results

**Figure 1:**
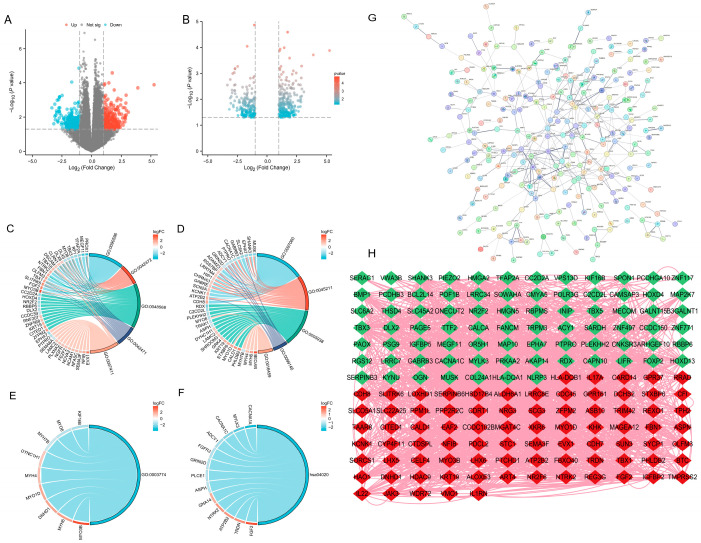
Identification and functional analysis of DEGs. (**A**,**B**) Volcano plots illustrating the distribution of differentially expressed genes (DEGs) between ASD and normal control samples in the GSE18123 blood microarray dataset. A total of 446 DEGs were identified, comprising 255 upregulated and 191 downregulated genes. (**C**–**F**) Gene Ontology (GO) enrichment analysis results of DEGs, including significant biological processes (e.g., regulation of membrane potential), cellular components (e.g., synaptic membrane), and molecular functions (e.g., gated channel activity). Representative enriched GO terms included GO:0042391, GO:0035637, and GO:0097060, among others (*p* < 0.05). (**G**) Protein–protein interaction (PPI) networks of DEGs generated by the STRING database, PPI enrichment *p*-value: <1.0 × 10^−16^. (**H**) Protein–protein interaction (PPI) networks of DEGs visualized using Cytoscape, illustrating the interaction landscape among key DEGs. Red and green indicate upregulated and downregulated genes. Number of nodes 172, Number of edges 1692. DEGs were identified with *p* < 0.05. Abbreviations: ASD, Autism Spectrum Disorder; DEGs, differentially expressed genes; GO, Gene Ontology; PPI, protein–protein interaction.

Through differential analysis of the GSE18123 autism and normal control blood microarray data, a total of (446) differentially expressed genes (DEGs) were identified, including (255) upregulated and (191) downregulated genes. The distribution of DEGs is illustrated in the volcano plot (Figure 1A,B). Certain genes, such as (HLA) and (CALCA), showed significantly altered expression between the two groups.

### 3.2. DEG Functional Enrichment Analysis and PPI Network Construction

GO enrichment analysis of the screened DEGs revealed that these genes were mainly involved in biological processes such as regulation of membrane potential, cellular components such as the synaptic membrane, and molecular functions such as gated channel activity. Specifically, significant GO terms included (GO:0042391, GO:0035637, GO:0097060 et. al) (*p* < 0.05). Detailed enrichment results are shown in Figure 1C–F and Table 1. A DEG protein–protein interaction (PPI) network was constructed using the STRING database and Cytoscape (Figure 1G,H).

### 3.3. CMap Drug Prediction Results and Biological Significance

**Figure 2:**
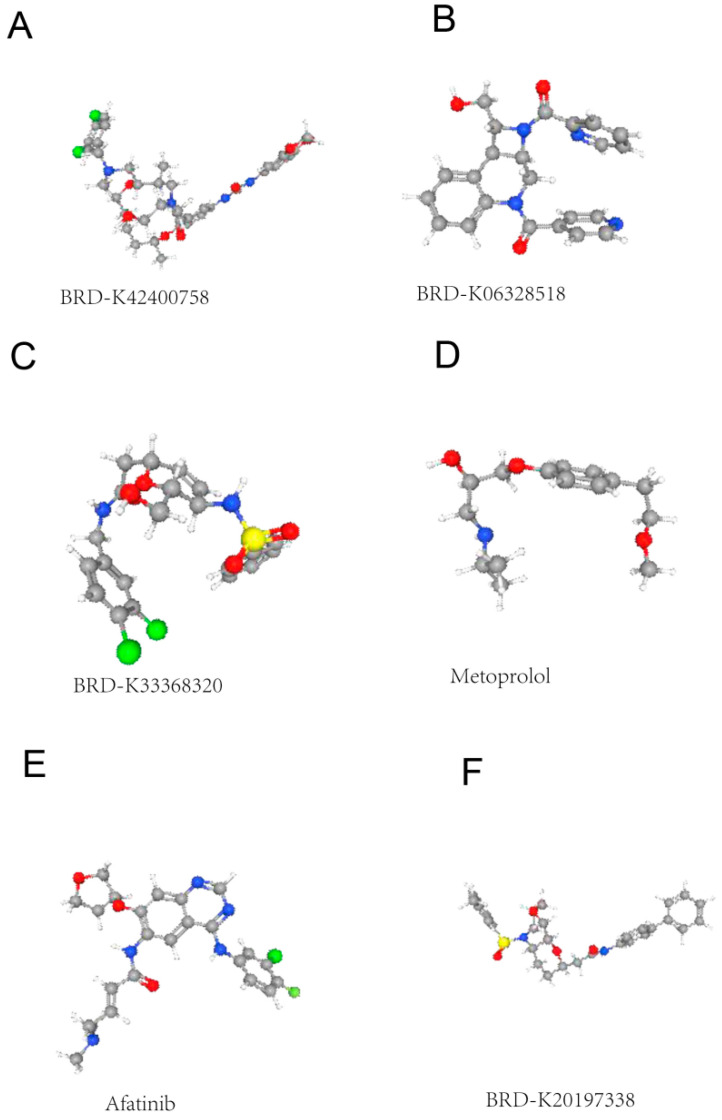
CMap-based prediction of small molecule compounds potentially targeting autism-related gene expression changes. (**A**) BRD-K42400758: A hydrophobic scaffold with pendant amide and amine groups. (**B**) BRD-K06328518: Aromatic core with nitro/oxo substituents. (**C**) BRD-K33663820: Complex heterocyclic framework with multiple heteroatoms. (**D**) Metoprolol: Beta-blocker scaffold shown to intersect with autism-relevant transcriptional programs in the dataset. (**E**) Afatinib: Epidermal growth factor receptor (EGFR) inhibitor-like structure. (**F**) BRD-K20197338: Linear amide-containing molecule.

Upregulated and downregulated DEGs were entered into the CMap website for drug prediction analysis. A total of (18,937) small molecule compounds were predicted to inversely match the DEG expression signatures. The most promising candidate drugs are listed in Table 2. These compounds may represent potential therapeutic targets for autism (Figure 2).

### 3.4. GeneCard Intersection Analysis

**Figure 3:**
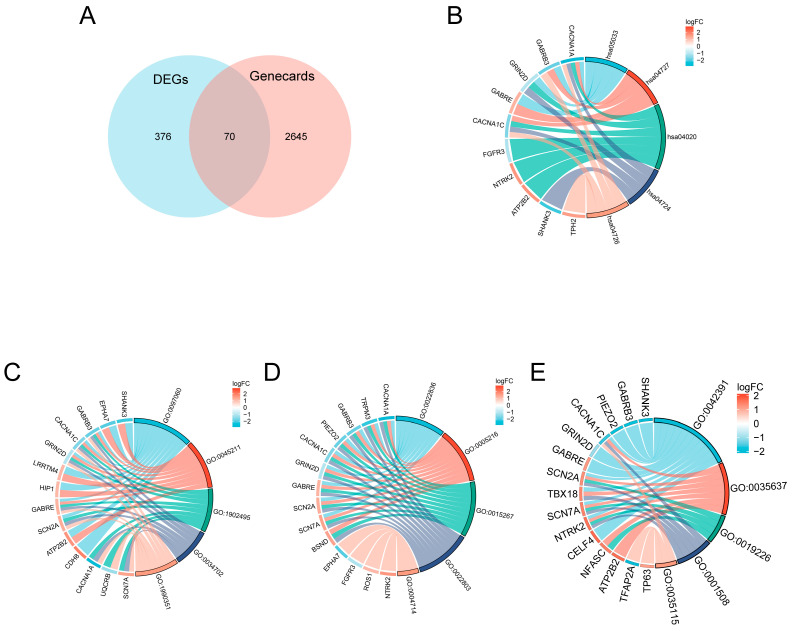
Intersection analysis of autism-related genes from GeneCard and DEGs, and functional enrichment. (**A**) Venn diagram illustrating the overlap between autism-related genes (GeneCard score > 10, *n* = 2715) and DEGs identified from the study. The intersection yielded 70 candidate genes. (**B**–**E**) Functional enrichment analysis of the intersecting genes, including Gene Ontology (GO) enrichment for biological process (BP), cellular component (CC), molecular function (MF), and Kyoto Encyclopedia of Genes and Genomes (KEGG) pathway analysis. Detailed enriched terms and pathways are shown.

GeneCard database retrieval of highly scored (>10) autism-related genes yielded 2715 candidate genes. Intersecting these with the DEGs resulted in 70 candidate genes. The screening process of intersection genes is displayed in the Venn diagram (Figure 3A). Figure 3B–E show further GO analysis, including the BP, CC, MF, and KEGG pathways.

### 3.5. Random Forest Selection of Top 10 Feature Genes

**Figure 4:**
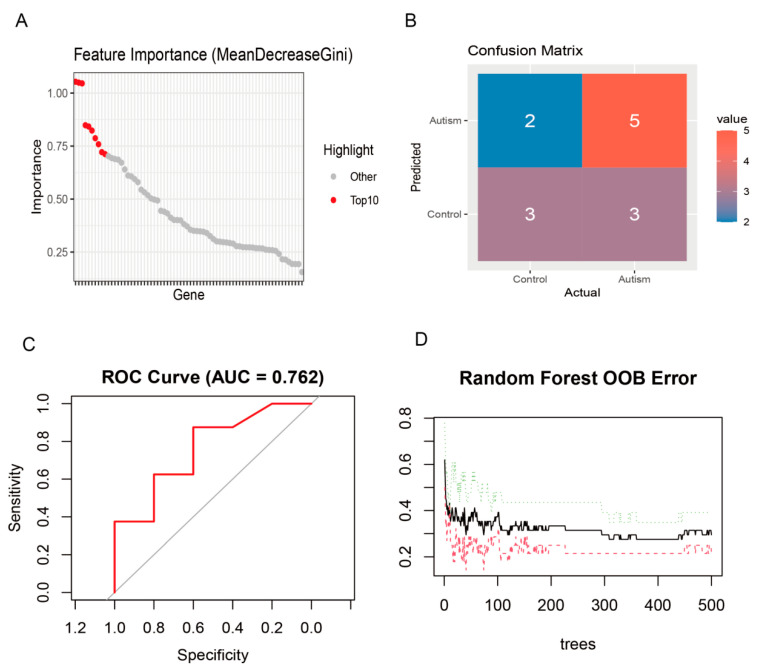
Identification of top 10 feature genes for autism prediction by random forest analysis. (**A**) Feature importance ranked by MeanDecreaseGini across all candidate genes; the top 10 contributors are highlighted in red, indicating a compact subset driving model decisions. (**B**) Confusion matrix summarizing predictions on the evaluation set, showing counts for Control and Autism classes and revealing both correct classifications and misclassifications. (**C**) ROC curve of the random forest classifier with an AUC of 0.762, reflecting moderate discriminative performance. (**D**) OOB error trajectory as a function of the number of trees; error stabilizes as the forest grows, supporting model robustness and generalization.

The random forest model identified a compact subset of genes that contributed most significantly to class separation. Based on OOB-derived importance scores, the highest-impact genes were concentrated in the top decile, from which the “Top 10” feature set emerged: *SHANK3*, *NLRP3*, *SERAC1*, *TUBB2A*, *MGAT4C*, *TFAP2A*, *EVC*, *GABRE*, *TRAK1*, and *GPR161*. This panel accounted for the largest cumulative decrease in node impurity across trees and dominated split decisions within the forest, indicating that these genes drive the model’s discrimination between Autism and Control. Performance evaluation on held-out data demonstrated stable generalization, with a validation ROC AUC of 0.762 and error rates consistent with OOB estimates, reinforcing the robustness of the selected feature set for predictive modeling (Figure 4A–D).

### 3.6. Immune Infiltration Analysis

**Figure 5:**
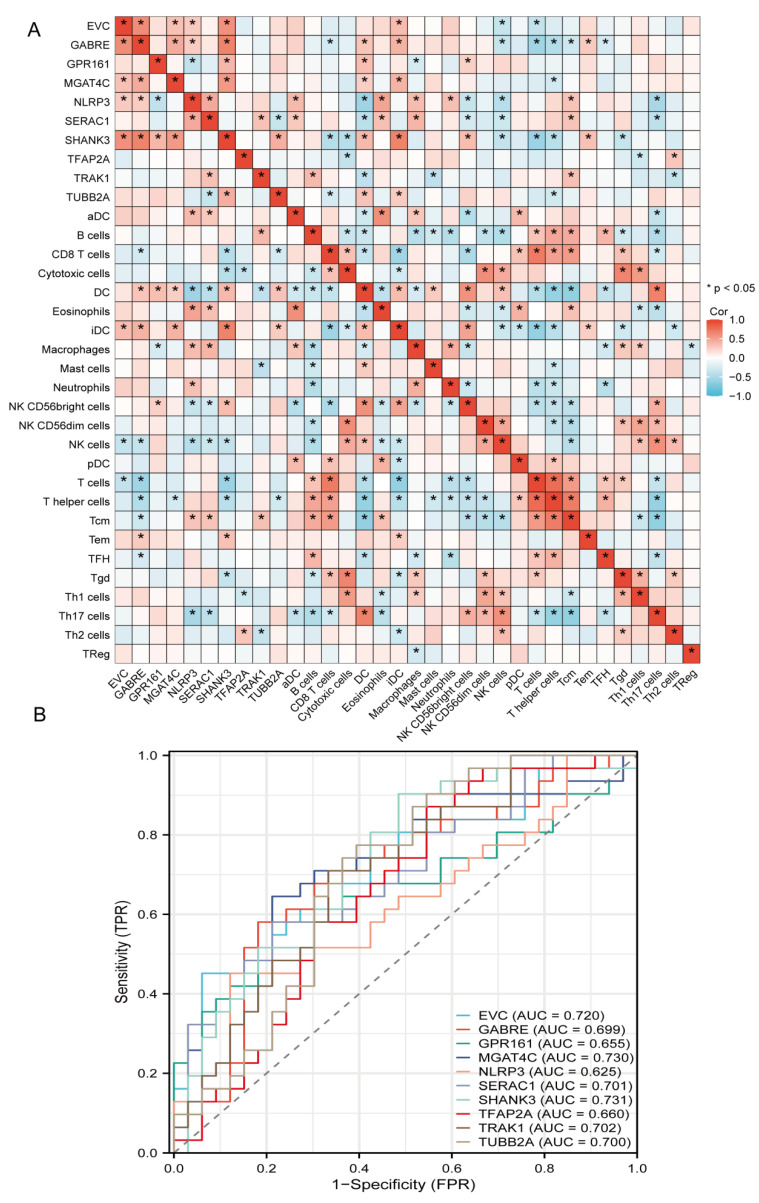
Immune infiltration correlation and diagnostic ROC analysis of key genes in autism. (**A**) Pairwise Spearman correlations between the top candidate genes and immune cell subsets. The heatmap reveals heterogeneous associations across innate and adaptive compartments; significant correlations are marked, suggesting coordinated regulation between specific genes and immune infiltration patterns. (**B**) Receiver-operating characteristic curves for ten marker genes assessed individually as diagnostic classifiers. Several genes reach acceptable-to-good performance with areas under the curve around or above 0.70. The strongest performer in this panel achieves an AUC near 0.73, while others cluster between approximately 0.62 and 0.71.

Correlation heatmap results revealed that the top 10 key genes exhibited significant correlations among various immune cell subpopulations (Figure 5A). The expression levels of immune-related genes *ISR1* and *ATP2B2* were closely associated with 13 distinct types of immune cells, demonstrating significant positive and negative correlations. Such dual-directional associations reveal complex pleiotropic effects within the immune microenvironment.

### 3.7. ROC Analysis of Top 10 Genes

ROC curve analysis evaluated the diagnostic potential of the top 10 key genes (Figure 5B). Most genes demonstrated robust discriminatory power with AUC values exceeding 0.70. MGAT4C exhibited the highest diagnostic accuracy (AUC = 0.730), while other significant performers included, *EVC* (0.720), *SERAC1* (0.701), *TRAK1* (0.702), and *TUBB2A* (0.700). *GABPB1* (0.699), *TFAP2A* (0.66), *GPR161* (0.655), and *NLRP3* (0.625) showed relatively lower discriminatory capacity. Collectively, these results indicate strong diagnostic utility for most key genes in distinguishing between clinical groups, with *GPR161* representing the most promising biomarker candidate (complete AUC data visualized in Figure 5B).

## 4. Discussion

ASD represents a clinically heterogeneous neurodevelopmental condition defined by persistent deficits in social communication, restricted behavioral repertoire, and stereotyped patterns of activity [23,24,25]. Contemporary diagnosis remains predicated on behavioral phenotyping, typically delaying intervention beyond critical neurodevelopmental windows [26,27,28].

Functional enrichment analysis demonstrated significant over-representation of DEGs in biological processes related to the regulation of membrane potential (GO:0042391, *p* = 2.85 × 10^−7^), multicellular organismal signaling (GO:0035637, *p* = 1.89 × 10^−6^), and transmission of nerve impulse (GO:0019226, *p* = 1 × 10^−4^). These findings bridge the gap between genetic susceptibility loci identified in GWAS studies and their functional consequences at the pathway level. Notably, the convergence of synaptic and immune pathways (e.g., complement activation in synaptic pruning) suggests potential mechanistic interplay that could explain the heterogeneity of ASD phenotypes. A study demonstrates the possible effect of transdermal nicotine when applied during waking hours in reducing aggression and improving sleep quality [29]. This suggests a possible association with nicotine addiction signaling pathways (hsa05033). The observed enrichment of calcium signaling pathways (hsa04020) may have particular therapeutic relevance, given the known role of calcium channel mutations in ASD [30]. Our GO/KEGG enrichment highlights ion channel and synaptic terms (GO:0042391, GO:0097060, GO:0022836, hsa04727 and hsa04020). These pathways provide a mechanistic bridge to recent evidence that fetal-to-early postnatal zinc–copper rhythmic dysregulation precedes ASD phenotypes [31]. Since Zn/Cu modulate channel gating and postsynaptic receptor function, disrupted elemental rhythms may desynchronize membrane potential control and neurotransmission, linking early-life metallomic dynamics to synaptic dysfunction observed in ASD.

Our CMap analysis predicted six compounds with significant negative enrichment norm_cs scores (<−1.5), including the beta-adrenergic blocker and the tyrosine kinase inhibitor. These findings align with recent clinical trials showing modest benefits of everolimus in ASD patients with neurodegenerative problems [32,33]. The predicted drugs predominantly target neuroinflammatory pathways rather than directly correcting synaptic defects, suggesting that combination therapies may be necessary for optimal outcomes. However, the translational potential of these predictions requires careful evaluation given the limited representation of neural cell types in the CMap reference database [34].

Machine learning models, especially random forests, are used extensively in microbiome research due to their ease of understanding, excellent performance, and incorporated feature selection (via estimating feature importance) [35]. Using random forest to screen characteristic genes and performing ROC diagnostic analysis demonstrated that these genes have good diagnostic efficacy for ASD [36,37]. Current ASD diagnostics rely solely on the behavioral phenotypes of toddlers, and such approaches are limited by the age wherein ASD can be reliably diagnosed. The ROC curve analysis indicated that these signature genes may also have diagnostic value for ASD.

Immune deconvolution analysis revealed significant elevation of iDC (*p* = 2.29 × 10^−9^) and regulatory T cells (*p* = 4.65 × 10^−7^) in ASD patients, consistent with recent single-cell RNA-seq studies of ASD brains [38]. The strong correlation between *SHANK3* expression and *iDC* (r = 0.66, *p* < 0.001) provides mechanistic support for the neuron–immune axis in ASD [39]. These findings should be interpreted in the context of methodological limitations, including the inherent noise in deconvoluting immune cell proportions from bulk RNA-seq data and potential confounding by peripheral inflammatory conditions unrelated to core ASD pathology.

In conclusion, this study analyzed ASD from the perspectives of drug prediction, signature gene screening, ROC diagnostic analysis, and immune infiltration, providing certain theoretical support for the diagnosis and treatment of ASD. However, further in-depth clinical research is still needed for practical application.

## Figures and Tables

**Table 1:** Identification and functional analysis of DEGs.

Ontology	ID	Description	Gene Ratio	Bg Ratio	*p* Value	*p* Adjust	Z Score
BP	GO:0042391	regulation of membrane potential	11/67	425/18,800	2.85 × 10^−7^	0.0004	0.30151
BP	GO:0035637	multicellular organismal signaling	7/67	164/18,800	1.89 × 10^−6^	0.0015	1.8898
BP	GO:0019226	transmission of nerve impulse	4/67	73/18,800	0.0001	0.0554	2
BP	GO:0001508	action potential	5/67	143/18,800	0.0002	0.0554	1.3416
BP	GO:0035115	embryonic forelimb morphogenesis	3/67	31/18,800	0.0002	0.0554	−0.57735
CC	GO:0097060	synaptic membrane	11/70	373/19,594	8.13 × 10^−8^	1.54 × 10^−5^	0.30151
CC	GO:0045211	postsynaptic membrane	9/70	271/19,594	5.05 × 10^−7^	4.77 × 10^−5^	−0.33333
CC	GO:1902495	transmembrane transporter complex	8/70	377/19,594	5.82 × 10^−5^	0.0033	−0.70711
CC	GO:0034702	ion channel complex	7/70	294/19,594	8.52 × 10^−5^	0.0033	−0.37796
CC	GO:1990351	transporter complex	8/70	399/19,594	8.64 × 10^−5^	0.0033	−0.70711
MF	GO:0022836	gated channel activity	9/65	340/18,410	2.89 × 10^−6^	0.0004	−1
MF	GO:0005216	ion channel activity	10/65	442/18,410	3.16 × 10^−6^	0.0004	−0.63246
MF	GO:0015267	channel activity	10/65	489/18,410	7.71 × 10^−6^	0.0005	−0.63246
MF	GO:0022803	passive transmembrane transporter activity	10/65	490/18,410	7.84 × 10^−6^	0.0005	−0.63246
MF	GO:0004714	transmembrane receptor protein tyrosine kinase activity	4/65	60/18,410	5.95 × 10^−5^	0.0030	0
KEGG	hsa05033	Nicotine addiction	4/38	40/8164	3.23 × 10^−5^	0.0038	−1
KEGG	hsa04727	GABAergic synapse	4/38	89/8164	0.0007	0.0301	−1
KEGG	hsa04020	Calcium signaling pathway	6/38	240/8164	0.0008	0.0301	−0.8165
KEGG	hsa04724	Glutamatergic synapse	4/38	114/8164	0.0018	0.0454	−2
KEGG	hsa04726	Serotonergic synapse	4/38	115/8164	0.0019	0.0454	−1

**Table 2:** CMap-based prediction of small molecule compounds.

No.	pert_id	pert_idose	norm_cs
1	BRD-K42400758	10 uM	−1.5654
2	BRD-K06328518	4 uM	−1.5366
3	BRD-K33368320	4 uM	−1.533
4	metoprolol	10 uM	−1.5314
5	afatinib	0.01 uM	−1.5266
6	BRD-K20197338	20 uM	−1.5233

## Data Availability

The original dataset (GSE18123) analyzed during this study is publicly available in the NCBI Gene Expression Omnibus (GEO) repository at https://www.ncbi.nlm.nih.gov/geo/query/acc.cgi?acc=gse18123, accessed on 6 August 2024. reference number [11]. Genecard -The Human Gene Database at https://www.genecards.org/ accessed on 10 March 2025. reference number [21].
